# Genotype-specific suppression of multiple defense pathways in apple root during infection by *Pythium ultimum*

**DOI:** 10.1038/s41438-018-0087-1

**Published:** 2019-01-01

**Authors:** Yanmin Zhu, Jonathan Shao, Zhe Zhou, Robert E. Davis

**Affiliations:** 1 USDA-ARS, Tree Fruit Research Laboratory, Wenatchee, WA 98801 USA; 2 USDA-ARS, Molecular Plant Pathology Laboratory, Beltsville, MD 20705 USA; 3 Research Institute of Pomology, Chinese Academy of Agricultural Science, Xingcheng, Liaoning 125100 P. R. China

**Keywords:** Transcriptomics, Plant genetics, Plant immunity

## Abstract

The genotype-specific defense activation in the roots of perennial tree crops to soilborne necrotrophic pathogens remains largely unknown. A recent phenotyping study indicated that the apple rootstock genotypes B.9 and G.935 have contrasting resistance responses to infection by *Pythium ultimum*. In the current study, a comparative transcriptome analysis by Illumina Solexa HiSeq 3000 platform was carried out to identify the global transcriptional regulation networks between the susceptible B.9 and the resistant G.935 to *P. ultimum* infection. Thirty-six libraries were sequenced to cover three timepoints after pathogen inoculation, with three biological replicates for each sample. The transcriptomes in the roots of the susceptible genotype B.9 were reflected by overrepresented differentially expressed genes (DEGs) with downregulated patterns and systematic suppression of cellular processes at 48 h post inoculation (hpi). In contrast, DEGs with annotated functions, such as kinase receptors, MAPK signaling, JA biosynthesis enzymes, transcription factors, and transporters, were readily induced at 24 hpi and continued up-regulation at 48 hpi in G.935 roots. The earlier and stronger defense activation is likely associated with an effective inhibition of necrosis progression in G.935 roots. Lack of effector-triggered immunity or existence of a susceptibility gene could contribute to the severely disturbed transcriptome and susceptibility in B.9 roots. The identified DEGs constitute a valuable resource for hypothesis-driven studies to elucidate the resistance/tolerance mechanisms in apple roots and validating their potential association with resistance traits.

## Apple orchards: Finding genes that fight infection

The discovery of genetic traits that make certain apple trees resistant to soil pathogens may help generate hardier crops. After apple orchards are replanted, some trees succumb to pathogens and fungi that have accumulated in the soil. To ensure the longevity of orchard sites, researchers want to clarify the genetic makeup of resistant trees. Yanmin Zhu at the United States Department of Agriculture in Wenatchee, US, and co-workers conducted comparative genetic analysis of two apple genotypes (one susceptible, one resistant) during infection with the plant pathogen *Pythium ultimum*. They found that the genotypes have multiple differentially-expressed genes. Key genes and signaling pathways in the susceptible tree’s roots were suppressed 48 h after infection when the tree was most vulnerable, whereas the resistant tree responded within 24 h, upregulating genes that strengthen roots and fight disease.

## Introduction

Apple replant disease (ARD) is a serious hindrance to the establishment of economically viable orchards on replant sites. ARD is caused by a pathogen complex primarily consisting of necrotrophic soilborne oomycetes (*Phytophthora* and *Pythium*) and fungi (*Ilyonectria* and *Rhizoctonia*)^1–3^. Among them, *Pythium ultimum* is a major component of this pathogen complex in orchard soil worldwide^2,4,5^. Pre-plant fumigation of orchard soils to eradicate ARD pathogens has been the primarily available control method for ARD^6^, but the currently available fumigants are under increasingly regulatory restriction due to environmental concerns. In addition to cost, the effects of fumigation are short-lived, and this method is not feasible after orchard establishment. Maximized exploitation of naturally existing resistance traits can offer a more cost-effective and durable control strategy for the management of soilborne diseases in tree fruit production systems. Currently, the conventional breeding for perennial tree crops with the aim of incorporating root resistance traits is a lengthy and resource-demanding process with poor predictability. This is due to the complex disease etiology of ARD, the perennial nature of apple as a tree crop, and the lack of reliable screening methods for root resistance to ARD. Elucidation of the molecular defense activation patterns and identification of key regulatory genes of resistance traits in apple roots could offer the opportunity of molecular marker assisted breeding, which will enhance the precision and efficiency for incorporating resistance traits into new apple rootstocks^7^.

Maximized immune output requires coordinated re-programming of cellular processes and efficient re-direction of metabolic activities in plant cells. Plants utilize a two-layer immune system to deter pathogen establishment and progression^8–10^. Plasma membrane embedded plant pattern recognition receptors (PRRs) can recognize the conserved pathogen-associated molecular pattern (PAMP) and activate so-called PAMP-triggered immunity (PTI)^11–13^. PTI is generally considered as a basal, nonspecific response^9,12^. However, adapted pathogens can suppress or bypass PTI through the secretion of evolved effector proteins^8,10,13^. On the plant side, co-evolved resistance (R) proteins directly or indirectly interact with effectors and initiate the second layer of defense, effector-triggered immunity (ETI)^10,11,14^. ETI leads to a stronger and more specific defense response toward those pathogen isolates that produce the recognized effector. These two layers of immune responses include multiple and often overlapping cellular processes, such as the spike of cytosolic Ca^2+^ concentration, production of reactive oxygen species (ROS), defense signaling transduction, defense hormone biosynthesis, generation and transport of secondary metabolites, callose deposition, and secretion of antimicrobial proteins^8,15–17^. Between genotypes within a species, the patterns of defense activation could determine the outcome of plant-pathogen interactions^18^.

Crosstalk between plant hormones plays a key role in tailoring specific and effective defense activation towards different types of attackers. Jasmonic acid (JA) and ethylene (ET) are well-known defense hormones in response to infection by necrotrophic pathogens. Various families of transcription factors (TFs) have been demonstrated for their critical roles of regulating defense activation in many pathosystems^19–21^. For example, JA-inducible R2R3-MYB (myeloblastosis oncogene), WRKY33 (containing signature WRKY amino acid residues) and ethylene responsive factors (ERFs) are essential in activating phenylpropanoid and terpenoid biosynthesis, and other defense-related pathways^22–24^. Specific to apple, phenolic compounds, such as phloridzin, are predominately accumulated in apple leaves in response to pathogen infection^25^. However, the underlying molecular mechanisms of genotype-specific defense activation patterns in apple roots toward infection by necrotrophic soilborne pathogens, are largely unexplored^26–29^. Comparative transcriptomic analysis can be employed to identify the differentially expressed genes (DEGs) in apple roots between resistant and susceptible apple rootstock genotypes as they are challenged with *P. ultimum*.

RNAseq-based transcriptome analysis has become a powerful tool for unraveling the global networks of transcriptional regulation^30–32^. Previous transcriptome profiling revealed the time course of global defense activation in apple roots using eight timepoints from 0 to 96 h post inoculation (hpi) by *P. ultimum*^27^. The defense response peaked at 48 hpi based on the number of identified DEGs. Recent phenotyping efforts demonstrated distinct resistance responses between two apple rootstocks, Bud 9 (B.9) and Geneva® 935 (G.935)^28^ to *P. ultimum* infection. In the current study, comparative transcriptomic analyses were performed using root tissues of equivalent developmental stages between these two genotypes that were simultaneously inoculated with the same preparation of *P. ultimum* inoculum (Figure S1). The objective was to identify the differentially regulated genes and pathways, which may contribute to the observed phenotypic variations between roots of G.935 and B.9 in response to *P. ultimum* infection. The identified DEGs will be a valuable resource for subsequent hypothesis-driven studies to functionally analyze their roles in genotype-specific defense activation and resistance phenotypes in apple roots.

## Results

A total of 426,001,826 paired-end reads of 150 bp were generated by Illumina Solexa HiSeq 3000 platform for 42 libraries, which cover two treatments (control and infected), three biological replicates and four timepoints for both genotypes (Figure S2). Results of data analyses for six libraries representing the transcriptome variations between two genotypes prior to pathogen inoculation at 0 timepoint were reported elsewhere^26^. This report analyzed the identified DEGs by comparing transcript abundance between mock-inoculated control root tissues and those from *P. ultimum* infected root tissues within each genotype. Based on the number of identified DEGs, the susceptible B.9 rootstock had a dramatically perturbated root transcriptome associated with *P. ultimum* infection (Table 1). In sharp contrast, a less disturbed transcriptome was observed in the root of the resistant G.935. The ratios between upregulated and downregulated DEGs between these two genotypes were revealing, especially at 48 hpi. Almost half of the DEGs identified from B.9 roots showed downregulated expression due to *P. ultimum* infection, but only 1 in 12 DEGs showed downregulation in G.935. A substantial number of genes downregulated in B.9 at 48 hpi suggested severely suppressed cellular processes from *P. ultimum* infection. The RNA-seq data was deposited in SRA (Sequence Read Archive) at the NCBI website under the accession number SRP117760 (ftp://ftp-trace.ncbi.nlm.nih.gov/sra/review/SRP117760).

**Table 1:** Number of differentially expressed genes (DEGs) identified at each of three timepoints in two genotypes during apple root response to *P. ultimum* infection

	*B.9*			*G.935*		
	24 hpi	48 hpi	72 hpi	24 hpi	48 hpi	72 hpi
Total DEGs	484	2309	1207	589	559	141
% of all apple genes (from apple genome v3.0.a1)	0.77	3.66	1.91	0.93	0.89	0.22
Upregulated DEGs	453	1147	1090	439	517	111
Downregulated DEGs	31	1162	117	150	42	30
Ratio of up- and downregulated	14.61	0.99	9.32	2.93	12.31	3.7
Uncharacterized (Nr)	70	412	163	88	60	20
% among all DEGs	14.5	17.8	13.5	14.9	10.7	14.2

The DEG numbers were calculated based on the analyses as described in M&M. The value of log_2_FC ≥ 1 and *p-*adj values ≤ 0.05 were used as selection criteria. *hpi* hours post inoculation, *Nr* NCBI non-redundant protein sequences

### DEGs encoding receptor kinases and mitogen-activated protein kinase (MAPK)

The roles of wall-associated kinases (WAKs) in plant immunity have been well documented in other pathosystems, such as rice blast^33,34^. In the present dataset, all but one identified WAK-encoding DEG was upregulated in the infected tissue compared with the respective mock inoculation control (Table 2). There were more WAK genes induced at the initial stage of 24 hpi in G.935 in comparison with B.9, which could suggest a quick response in this resistant genotype. Noticeably, less than one third of the identified DEGs were the conserved genes between two genotypes.

**Table 2:** Differentially expressed genes (DEGs) encoding wall-associated kinases (WAKs) during infection by *Pythium ultimum*

	log_2_FC per genotype and timepoint					
Genes	B.9-24	B.9-48	B.9-72	G.935-24	G.935-48	G.935-72
*MDP0000230524*	2.0			1.2	1.7	
*MDP0000186304*	1.3		1.7		1.7	
*MDP0000183195*		1.9				
*MDP0000281090*		1.8	1.2			
*MDP0000426154*		2.2	1.4			
*MDP0000251865*		1.8				
*MDP0000656197*		1.5				
*MDP0000170906*		1.7	1.3			
*MDP0000567084*		2.0	1.2	1.4	2.3	
*MDP0000317025*		3.9				
*MDP0000153539*		3.3	1.7		1.6	
*MDP0000206106*			1.3			
*MDP0000267001*			3.4	1.6	2.8	
*MDP0000562934*				−1.2		
*MDP0000681106*				1.0		
*MDP0000240979*				1.0		
*MDP0000247933*				1.0		
*MDP0000236093*					*2.1*	

The values of log_2_FC ≥ 1 and *p-*adj values ≤ 0.05. were used as selecting criteria of DEGs using the method described in MM. The symbol “−” in front of the number indicates the downregulation in infected root as compared with mock-inoculation control

**Fig. 1:**
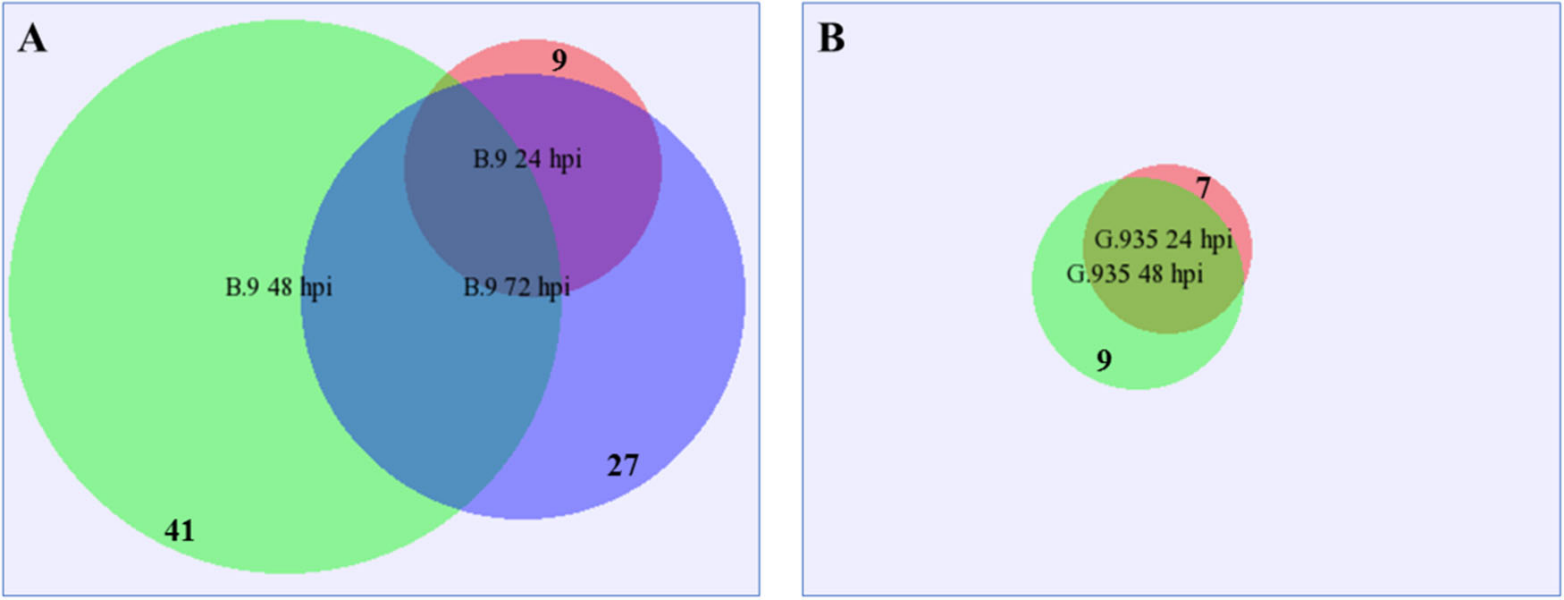
Proportional Venn diagram showing identified differentially expressed genes (DEGs) encoding lectin RLKs per genotype and timepoint. **a** Identified lectin RLK from B.9 genotype; **b** Identified lectin RLK from G.935 genotype. DEGs from each timepoint and genotype was visualized by the size of circle and with the numbers listed on the edge of the circle; the overlapped area indicated the identical genes between timepoints

Lectin receptor like kinases (RLKs) are a large group of cell surface receptors, which have been implicated in many biological processes including defense activation^35,36^. Almost all identified lectin RLK DEGs in this study were upregulated with a few exceptions (Fig. 1). A comparable number of DEGs were identified between the two genotypes at 24 hpi, yet a significant difference was observed at 48 and 72 dpi. About three times more lectin RLK DEGs were identified in B.9-48 than in G.935-48. Within the B.9 sample series, all genes except one were identical between 24 and 72 hpi, but many DEGs encoding additional lectin RLK (green circle in Fig. 1a) were specific to the B.9-48 samples. A more consistent upregulation of lectin RLK DEGs was observed in G.935 roots from 24 to 48 hpi, though no DEG was identified in the G.935-72 sample. A highly-expressed MDP0000228426 was one of only two downregulated lectin RLKs encoding genes in B.9-48. Several additional categories of protein kinase-encoding genes were differentially regulated in response to *P. ultimum* infection. About one third to half of the DEGs were downregulated in B.9-48 sample (Table 3).

**Table 3:** Differentially expressed genes (DEGs) in other categories of receptor kinases by genotype and timepoint

	Receptor-like protein kinase	LRR receptor-like serine/threonine-protein kinase	Cysteine-rich receptor-like protein kinase	CBL-interacting serine/threonine protein kinase	Unclassifiable receptor/kinase only for B.9
B.9-24	+4/0	0/−1	+1/0	+1/0	+2/−1
B.9-48	+13/−4	+8/−9	+5/−3	+5/−4	+13/−9
B.9-72	+14/0	+3/0	+6/0	+/0	+10/−3
G.935-24	+5/−1	+1/−1	+1/0	0/0	NA
G.935-48	+3/0	+3/0	+1/−1	+1/0	NA
G.935-72	0/0	0/0	0/0	0/0	NA

Values represent the numbers of DEGs, which are annotated as kinase receptors of various categories. “ + ” stands for upregulated expression pattern; and “−” stands for downregulated expression patterns at specific timepoints for both genotypes.

**Fig. 2:**
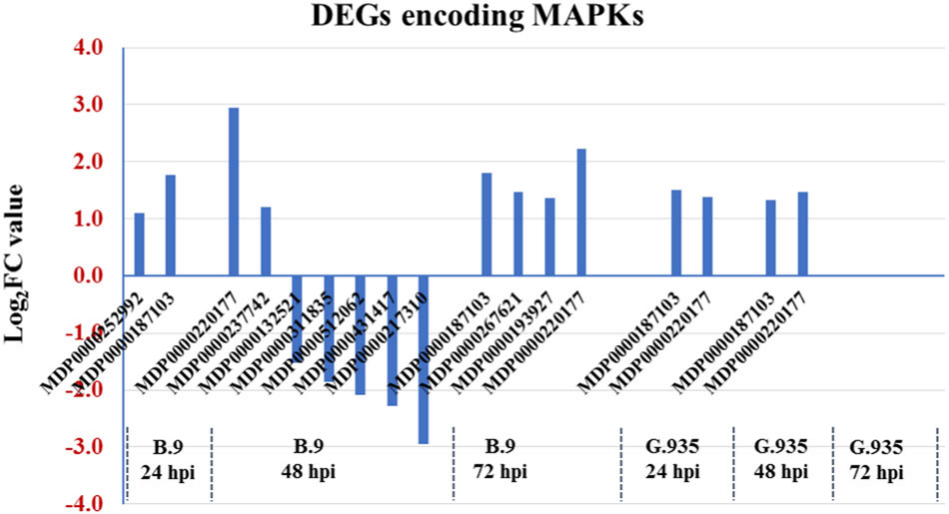
Genotype-specific regulation patterns of mitogen-activated protein kinases (MAPK) encoding genes in apple roots during *Pythium ultimum* infection. Numbers on *Y* axis are the log_2_FC values, which represent the expression changes by comparing those from *P. ultimum* infected tissues and mock-inoculated control tissues. The positive values indicate the upregulation due to pathogen infection, whereas the negative values represent the downregulated expression. Identified genes were listed on *X* axis by genotype and timepoint

The MAPK mediated signal transduction cascade is known to be essential during defense activation in response to pathogenic pressure^37–39^. The genotype-specific regulation patterns for MAPK encoding DEGs represent a “typical” trend observed for several functional groups in this dataset (Fig. 2). In resistant G.935 roots, two genes were consistently upregulated at 24 to 48 hpi, with no DEGs identified at 72 hpi. A contrasting regulation scheme was observed in susceptible B.9 roots: five out of seven MAPK encoding DEGs were downregulated in the B.9-48 sample, though all four identified DEGs were upregulated in the B.9-72 sample, most of them were newly induced genes. These observations seemed to indicate that factors from *P. ultimum* forced a transcriptional shift specifically in susceptible B.9 roots. MDP0000187103, which encodes a “mitogen-activated protein kinase kinase kinase 3-like” was consistently upregulated from 24 to 48 hpi in G.935. The same gene was upregulated at 24 and 72 hpi, however, its induction was interrupted specifically at 48 hpi in B.9 roots.

### DEGs encoding disease resistance proteins

**Fig. 3:**
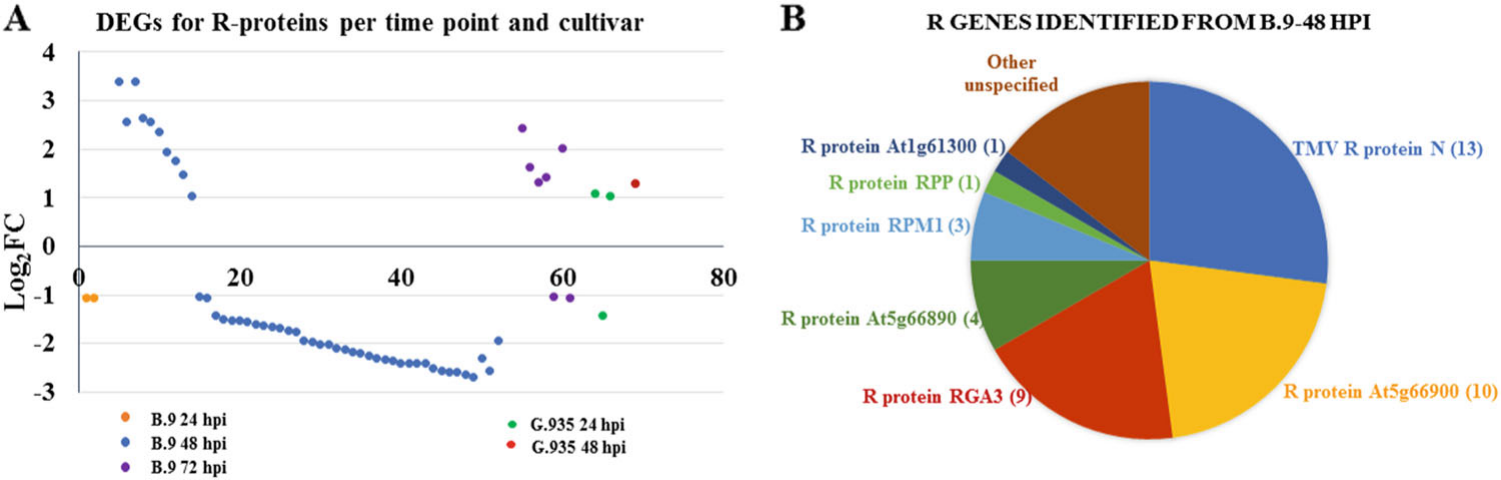
Differentially expressed genes (DEGs) encoding resistance (R) proteins due to *Pythium ultimum* infection. **a** The distribution and regulation patterns of identified R-genes per timepoint and genotype. Each colored dot represents an individual R-gene. Numbers on the *Y* axis are the log_2_FC values based on the comparison of expression levels between *P. ultimum* infected root tissues and mock-inoculated control root tissues. The positive values indicate upregulation and negative values indicate downregulation. **b** The pie chart illustrates the classes of proteins encoded by the *R*-genes in B.9-48, with numbers in parentheses for respective groups

The regulation patterns of resistance genes (*R-*genes) further exemplify the contrasting transcriptome changes between these two genotypes in response to *P. ultimum* inoculation (Fig. 3). Most of the DEGs encoding *R*-genes were identified in B.9-48. An overwhelming majority, 38 out of 48, were downregulated in the B.9-48 library (Fig. 3a). From other samples (genotype/timepoint combinations) only a small number (1–5) of DEGs were identified, and most of them were upregulated. Those identified *R*-genes from B.9-48 can be categorized into a few dominant groups based on their functional annotations (Fig. 3b). Three major groups, “TMV resistance protein N-like”, “disease resistance protein At5g66900” or “disease resistance protein RGA3”, contain 13, 10, and 7 genes, respectively. Notably, MDP0000138927 encodes a “protein SUPPRESSOR OF npr1-1, CONSTITUTIVE1-like” (SNC), which is known for its role in resistance to both bacterial and fungal pathogens^40^. Additionally, two DEGs (MDP0000134649 and MDP0000191848) encoding homologs of well-characterized susceptibility gene MLO^41^ were consistently upregulated to a high expression level in B.9 roots. In contrast, a different gene annotated as MLO homologous was only upregulated in G.935-24.

### DEGs encoding enzymes of defense hormone biosynthesis

**Fig. 4:**
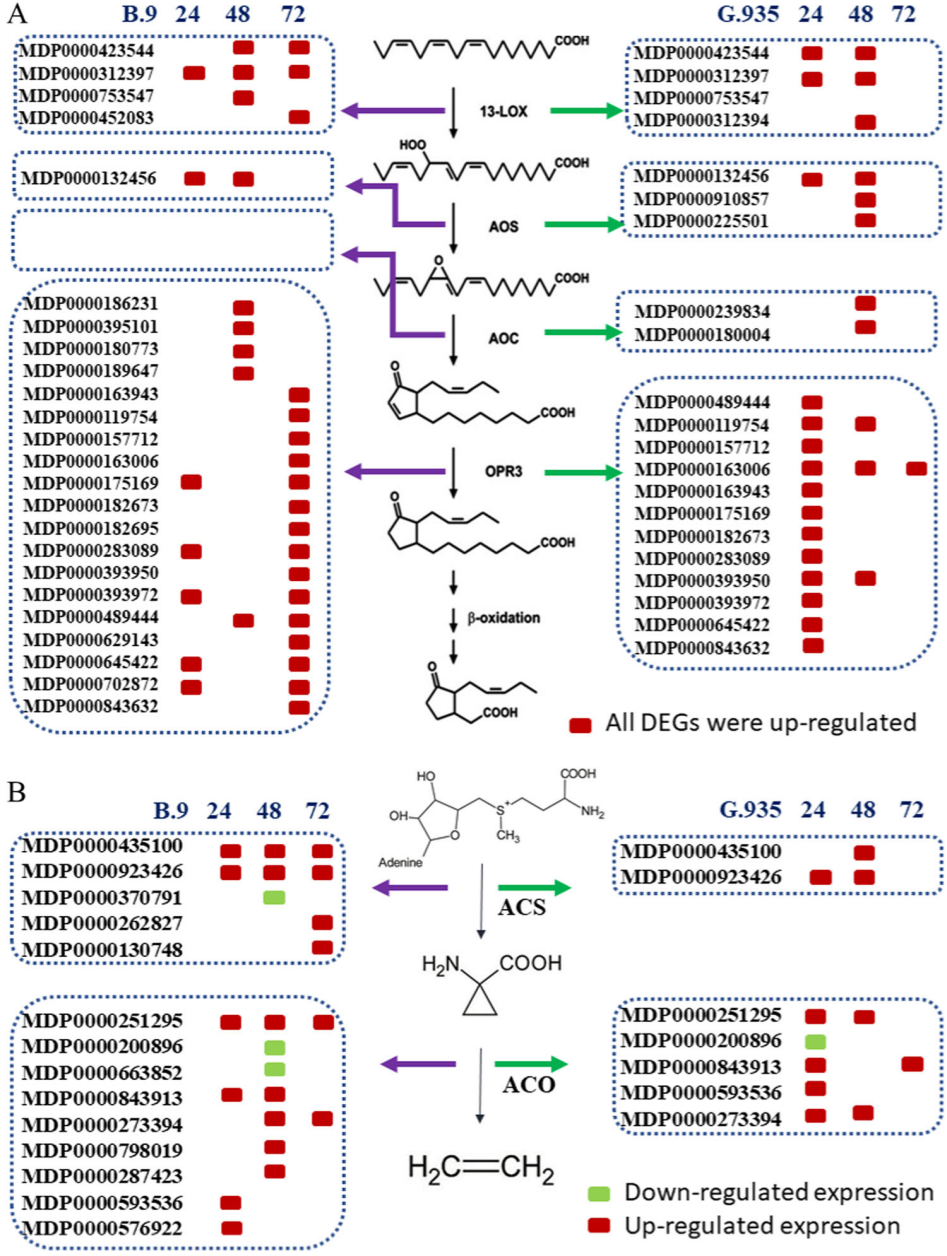
Differentially expressed genes (DEGs) encode enzymes in the ethylene and jasmonic acid biosynthesis pathways. **a** Identified genes functioning jasmonic acid (JA) in biosynthesis pathways by genotype and timepoint. **b** Identified genes functioning in ethylene (ET) biosynthesis pathways by genotype and timepoint

The crucial roles of JA and ET as defense hormones in response to infection by necrotrophic pathogens have been well-demonstrated in many pathosystems^15,17,42^. The systematic induction of JA and ET biosynthesis pathways appeared to be one of the most distinguishable transcriptome changes in *P. ultimum* infected apple roots. Genes encoding enzymes for the first four steps of the JA biosynthesis pathway^43^ were systematically upregulated with genotype-specific activation patterns (Fig. 4a). Two lipoxygenase (LOX) genes, MDP0000452083 and MDP0000423544, were induced in G.935 earlier than in B.9 roots. One allene oxide synthase (AOS) encoding gene, MDP0000132456, was shown to be consistently upregulated at 24 and 48 dpi in both genotypes. However, two extra AOS genes were exclusively identified from G.935 root at 48 hpi. In a previous transcriptome survey, MDP0000132456 was also identified as an upregulated AOS gene to *P. ultimum* infection^27^. Two allene oxide cyclase (AOC) genes, MDP0000239834 and MDP0000180004, were exclusively identified in G.935-48. The expression patterns of multiple DEGs encoding “oxophytodienoate reductases” (OPR) demonstrated an even more revealing aspect of JA biosynthesis between B.9 and G.935. A total of 12 encoding genes were upregulated in the G.935 root at 24 hpi, in contrast to five that were upregulated in B.9-24. Until 72 hpi, the same set of genes, which were induced in G.935-24, showed upregulation in B.9 root. These observations demonstrated that in B.9 roots the activation of OPR3 gene family was delayed by 48 hpi compared with the one in G.935.

Two genes encoding 1-aminocyclopropene-1-carboxylate synthase (ACS) (MDP0000435100 and MDP0000923426), which catalyzes the first step of ET biosynthesis pathway^44^, were consistently upregulated in both genotypes at 24 and 48 hpi. At 72 dpi their upregulations were continued plus two additional ACS genes, but only in susceptible B.9 roots (Fig. 4b). Multiple DEGs encoding 1-aminocyclopropane-1-carboxylate oxidase (ACO), the enzyme catalyzing the second step of ET biosynthesis pathway, were upregulated, and the genes were identical between the two genotypes. Genes participating in the metabolism and homeostasis of other plant hormones, including auxin, cytokinin, and strigolactone, appear to be an integral part in fine-tuning the defense activation in this pathosystem of apple roots interacting with *P. ultimum* (Table S1).

### DEGs encoding TFs

**Fig. 5:**
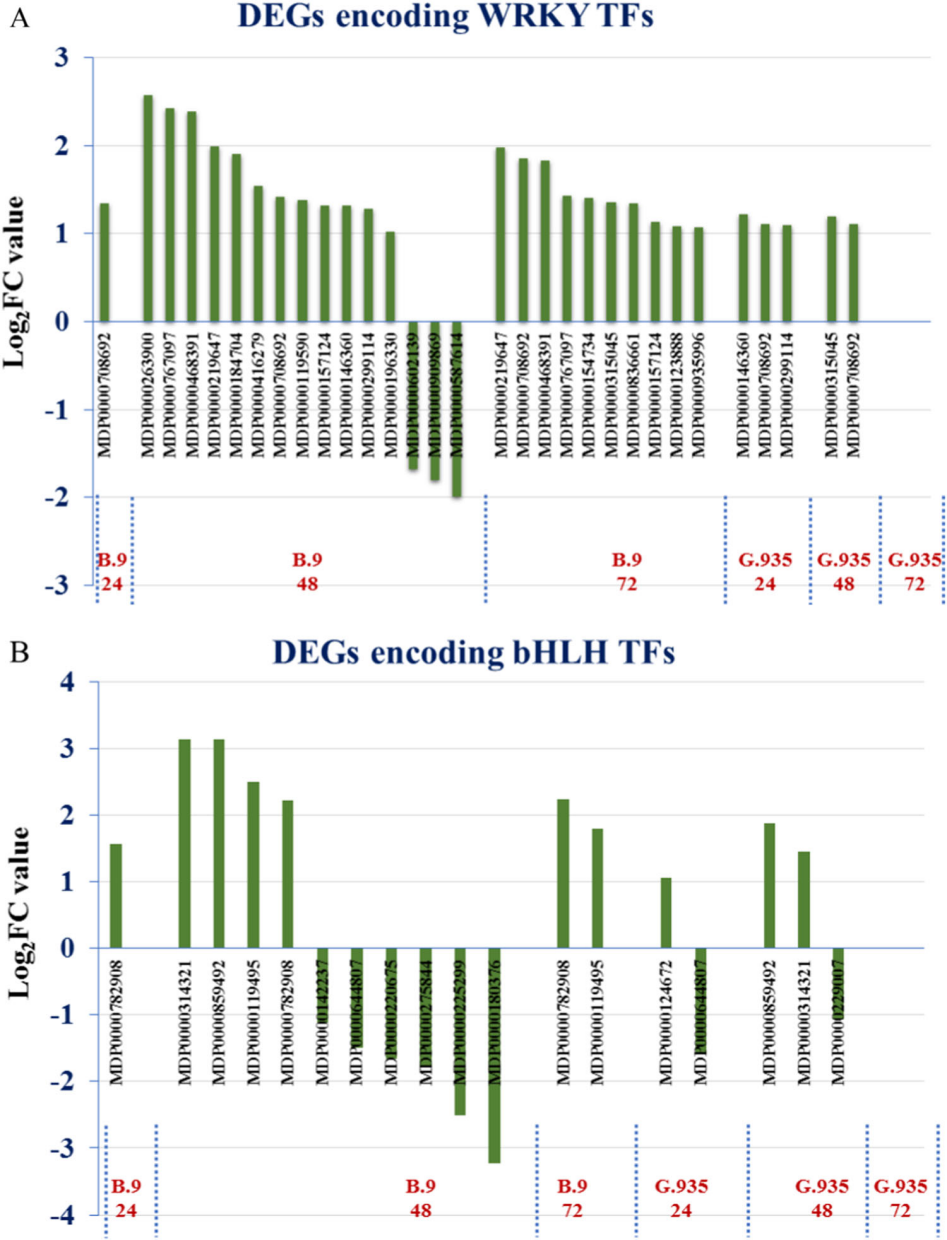
Genotype-specific regulation patterns of differentially expressed genes (DEGs) encoding WRKY (containing WRKY amino acids signature) and bHLH (basic helix-loop-helix) transcription factors. Values on the *Y* axis are the log_2_FC values representing the gene expression changes between *P. ultimum* infected root tissue and the mock-inoculated control tissue. The positive values represent upregulation and negative values represent downregulation of gene expression. Genes on the *X* axis are those identified at various timepoints in both genotypes

**Fig. 6:**
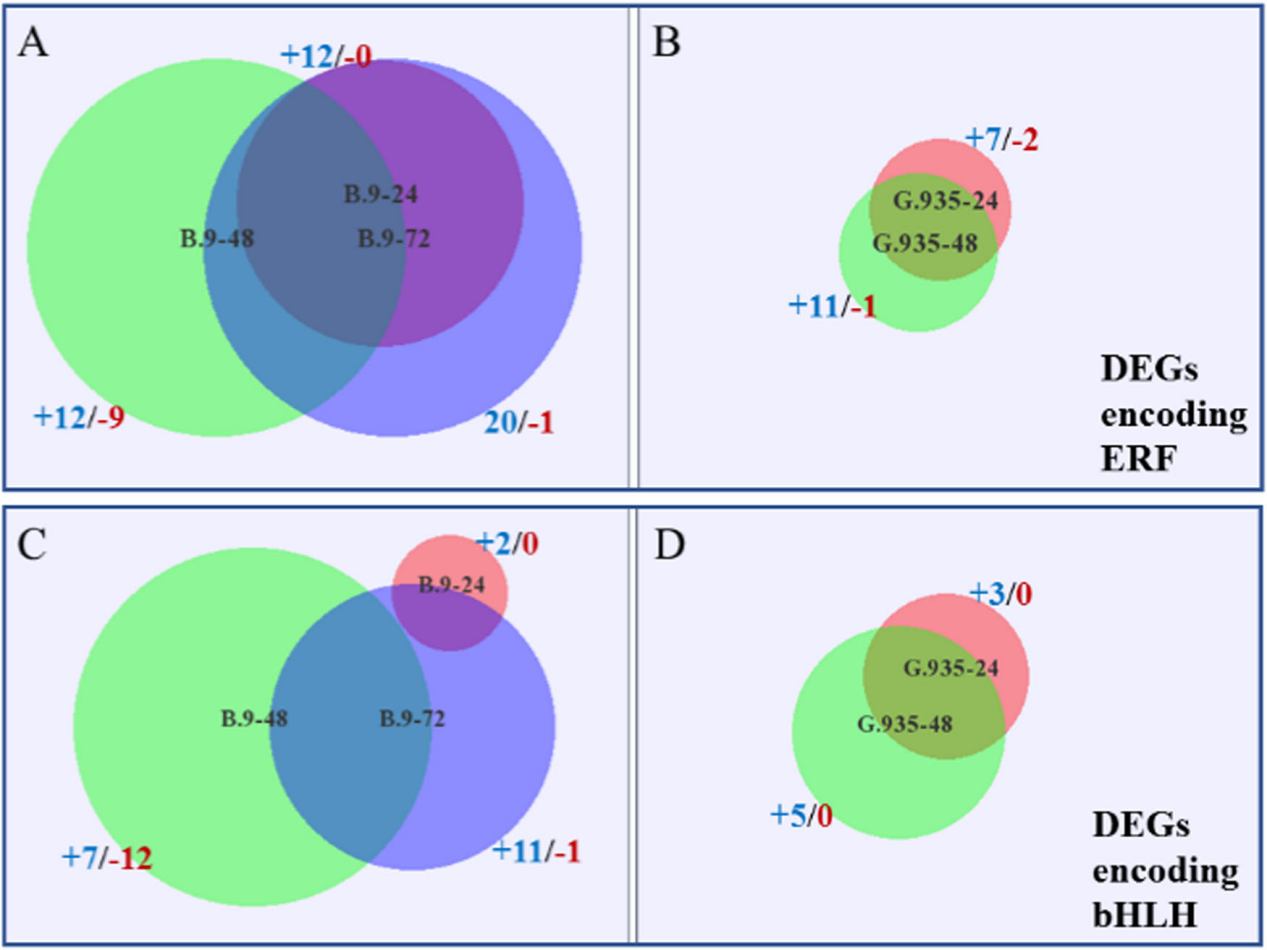
Differentially expressed genes (DEGs) encoding ERF (ethylene response factor) and MYB (myeloblastosis oncogene) transcription factors per timepoint and genotype. **a** Identified DEGs encoding ERFs in B.9; **b** Identified DEGs encoding ERFs in G.935. **c** Identified DEGs encoding MYB in B.9. **d** Identified DEGs encoding MYB in G.935. The number of identified DEGs from each timepoint and genotype were represented by the size of the circles; and the first number with “+” sign indicates those with upregulated patterns, the second number with “−” sign indicates those with downregulated expression patterns. The overlapped areas represent the identical genes between timepoints

The roles of TFs in plant defense activation toward infection by necrotrophic pathogens are well elucidated^19,22^. From the dataset, genes encoding four major TF families were differentially expressed between the two genotypes when challenged with *P. ultimum*. All identified DEGs encoding WRKY TFs were upregulated in B.9 samples except three of them in B.9-48, while the smaller subset of the genes identified in G.935 samples were all upregulated (Fig. 5a). MDP0000708692, a highly expressed gene encoding a TF homolog of Arabidopsis “WRKY transcription factor-33” was identified consistently in all samples except G.935-72. A few (1–3) bHLH (basic helix-loop-helix) encoding DEGs were identified per genotype/timepoint, except that there were ten DEGs in B.9-48, though six of them were downregulated (Fig. 5b). Ethylene response factors (ERFs) are well known for their role of regulating plant defense responses by integrating signals from both ET and JA^19^. Between the two genotypes, five DEGs encoding AP2/ERF, MDP0000323780, MDP0000299277, MDP0000127134, MDP0000122665, and MDP0000167207, were consistently upregulated at all timepoints except G.935-72 (Fig. 6a, b). Additional ERF-encoding genes were identified from B.9 roots, but about half of them were downregulated in the B.9-48 sample. Similar to the ERF TF family, a higher number MYB-encoding DEGs were identified in B.9 roots in response to *P. ultimum* infection (Fig. 6c, d). A new subset of MYB genes (represented by the purple area in Fig. 6c) were upregulated specifically in B.9-72, possibly in response to the suppression of MYB gene family members in B.9-48. In contrast, all identified DEGs encoding MYBs in G.935 were upregulated from 24 to 48 hpi.

### DEGs encoding enzymes of secondary metabolisms

**Fig. 7:**
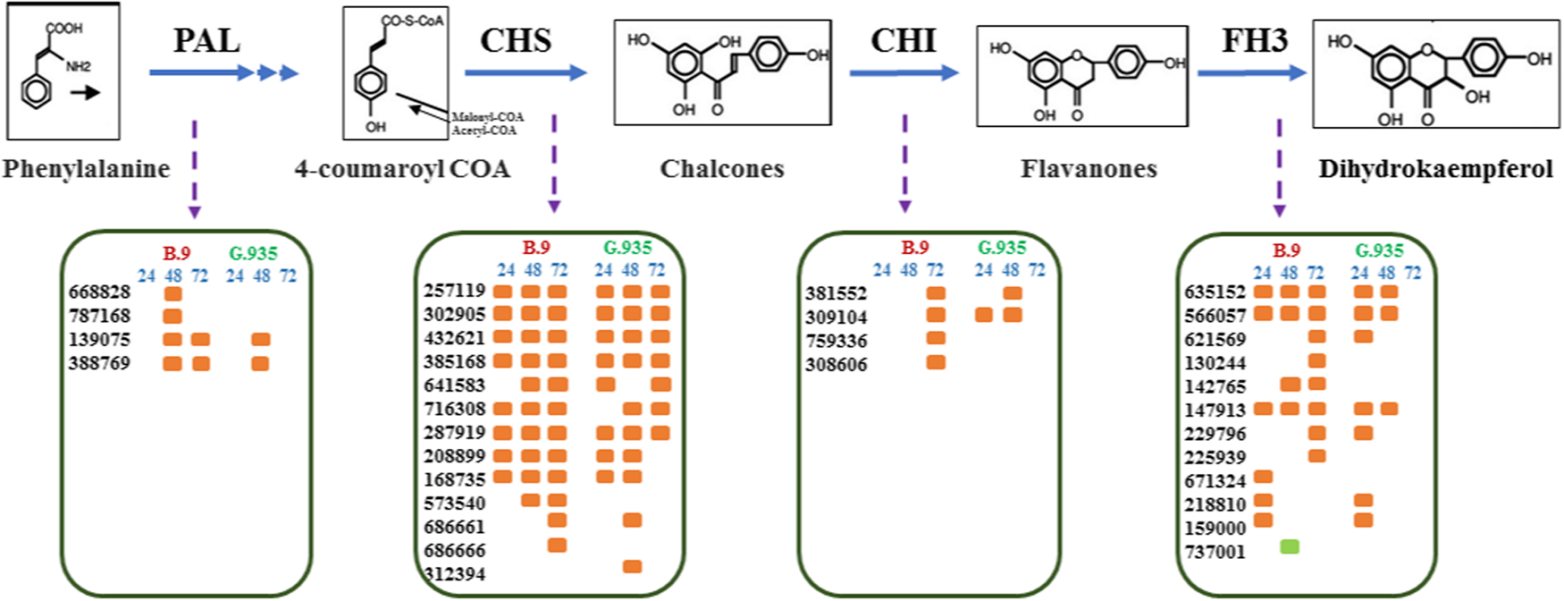
Differentially expressed genes (DEGs) with annotated functions at the early steps of flavonoid biosynthesis pathway. PAL phenylalanine ammonia-lyase, CHS chalcone synthase, CHI chalcone isomerase, FH3 flavanone 3-hydroxylas. The six-digit numbers are gene identifiers without MDP0000 prefix. Orange colored dots indicate the upregulated patterns following *P. ultimum* infection; green colored dot indicates the downregulated patterns

DEGs encoding enzymes functioning at the early steps of the flavonoid biosynthesis pathway were systematically upregulated (Fig. 7). These enzymes include phenylalanine ammonia-lyase (PAL), chalcone synthase (CHS), chalcone isomerase (CHI), and flavonol synthase/flavanone3-hydroxylas (F3H). For most of these gene families, identical genes and comparable activation patterns were identified across timepoints and genotypes. One exception was for genes encoding CHI, where a delayed induction was observed in the roots of susceptible B.9, as compared to the same genes in G.935. Several other gene families encoding enzymes that catalyze the other branches of secondary metabolism (Table 4), including caffeic acid 3-O-methyltransferase (COM), squalene monooxygenase, geraniol 8-hydroxylase, and UDP-glycosyltransferase (UGT), were mostly upregulated at each timepoint-genotype, except B.9-48. Among these gene families, the larger number of UGT-encoding and COM-encoding genes in G.935-24 were upregulated compared with those in B.9-24. Such regulation patterns of earlier induction resemble those for several other gene families, such as WAK, WRKY, AOS, and OPR.

**Table 4:** Differentially expressed genes (DEGs) encoding enzymes functioning at other sections of the secondary metabolism pathways

	B.9-24	B.9-48	B.9-72	G.935-24	G.935-48	G.935-72
Caffeic acid 3-O-methyl transferase	1	12	9	3	3	3
Squalene monooxygenase	2	3 (−2)	4	2	2	0
Geraniol 8-hydroxylase	0	3 (−3)	2	0	0	0
UDP-glycosyl transferase	3	18 (−1)	16	8 (−1)	11	2

The values represent the numbers of identified DEGs per timepoint and genotype. Numbers with “−” sign in parentheses represent the downregulated genes among the total number of identified DEGs for a specific timepoint

### DEGs encoding transporters

**Fig. 8:**
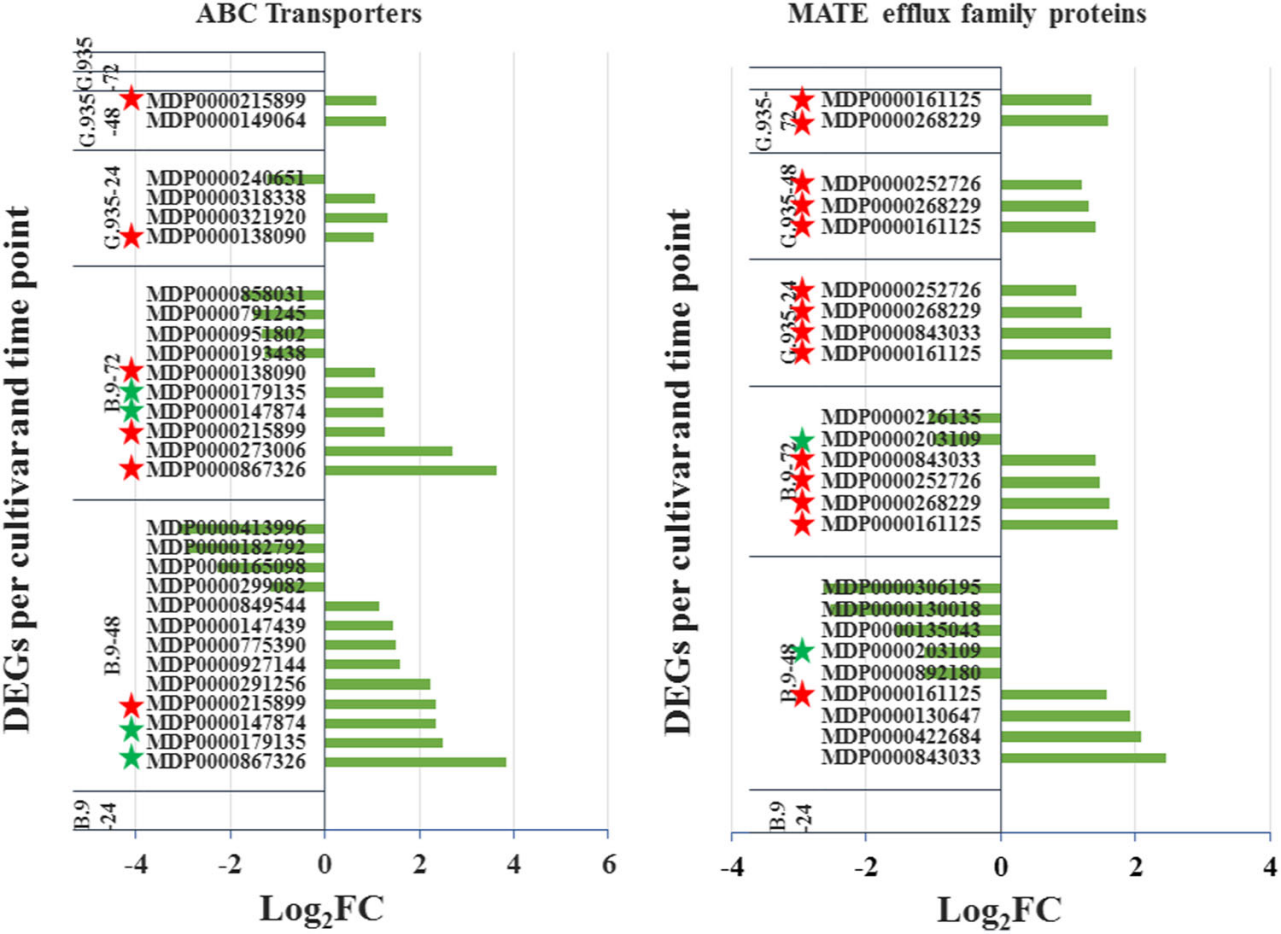
Differentially expressed genes (DEGs) encoding ABC transporters and MATE efflux family protein. Individual genes are listed along the *Y*-axis per timepoint and genotype. Numbers on the *X*-axis represent the log_2_FC value based on the comparison of the transcript levels between mock-inoculated control and *P. ultimum* infected tissues. Red stars denote the consistent genes between genotypes and timepoints. Green stars indicate the consistency between the timepoints within the same genotype

DEGs encoding ABC transporter and MATE (multidrug and toxic compound extrusion) efflux family proteins clearly demonstrated the genotype-specific regulation patterns. A few similar regulation features were observed for both gene families (Fig 8). (1) Earlier induction for these genes at 24 hpi was identified in G.935 roots, but not in B.9. (2) In the B.9-48 sample, 13 and 10 DEGs were identified for both gene families, respectively, but multiple genes were downregulated. On the other hand, a smaller number of DEGs were identified in G.935-48 and all were upregulated. (3) Specific for DEGs encoding “MATE efflux family protein”, identical genes were consistently upregulated at all three timepoints in G.935, while the same genes were only upregulated until 72 hpi in B.94 (denoted by red stars). Among DEGs encoding ABC transporters, identical genes were rare between genotypes and timepoints. In addition, genes encoding several other families of transporters were also differentially regulated in response to *P. ultimum* infection (Table 5), including those encoding “nitrite transporter”, “phosphate transporter”, “potassium transporter”, “sulfate transporter”, “lysine/histidine transporter”, and “bidirectional sugar transporter SWEET1”.

**Table 5:** Differentially expressed genes (DEGs) encoding proteins functioning as transporters

Transporter families	DEGs per genotype and timepoint											
	B.9-24		B.9-48		B.9-72		G.935-24		G.935-48		G.935-72	
	+^a^	−^b^	+	−	+	−	+	−	+	−	+	−
Nitrite transporter	2	1	4	1	3	2	3	1	1	2	1	
Phosphate transporter	1		4	1	1	1	3		2			
Potassium transporter		6		3								
Sulfate transporter					3				3			
Lysine/histidine transporter	2		1	3	2		3		3			
Sugar transporter SWEET1			4		5		4	1	3		1	

The numbers of genes identified at each timepoint were based on comparison of normalized expression levels between mock-inoculated tissue and *P. ultimum* infected tissues

^a^+Denote the gene was upregulated

^b^−Denote the gene was downregulated

### DEGs encoding pathogenesis-related (PR) proteins and other defense-related proteins

DEGs encoding PR proteins and other proteins with the proposed functions in basal immune responses constitute a substantial part of apple root transcriptome changes in response to *P. ultimum* infection. The identified DEGs encoding all three classes of PR-proteins, i.e., those encoding “chitinase”, “thaumatin”, and “PR-4”, were relatively comparable between the two genotypes (Table 6), except for a slightly larger number of genes for “thaumatin” in G.935-24 and “chitinase” in B.9-24. DEGs encoding five classes of proteins including “patatin”, “laccase”, “mandelonitrile lyase”, “germin”, and “nudix hydrolase”, which could function in the processes of releasing antimicrobial chemicals for restricting pathogen progression, were all upregulated with rare exception. DEGs encoding five classes of oxidases were mostly upregulated in response to *P. ultimum* infection, except for the fact that a large number of “L-ascorbate oxidase” and “protein SRG1” encoding genes were downregulated in B.9-48. For those gene families encoding various oxidases, more DEGs were identified in B.9 than G.935 at each timepoint. All or most of the identified DEGs for “pectin esterase inhibitor” and “callose synthase” were specifically downregulated in B.9-48. For a few gene families including “germin”, “protein SRG1”, “nudix hydrolase”, and “pectin esterase inhibitor”, the larger number of upregulated DEGs were identified in G.935-24 than that in B.9-24.

**Table 6:** Differentially expressed genes (DEGs) encoding PR proteins and other proteins of basal immune response

Protein categories	B.9-24*	B.9-48	B.9-72	G.935-24	G.935-48	G.935-72
Chitinase	13	11	17	8	11	0
PR-4	2	2	2	1	2	0
Thaumatin	1	1	5	3	5	0
Patatin	15	20	17	13	18	1
Laccase	6	7	6	7	4	1
Germin	2	8	8	6	5	0
Nudix hydrolase	1	6 (−2)	0	2	2	0
Mandelonitrile lyase	5	9	7	3	3	1
Protein SRG1	2	11 (−9)	7	4	3	0
L-ascorbate oxidase	0	8 (−4)	5	0	3	0
Peroxidase	10	12	19	4 (−1)	6	1
Lignin-forming anionic peroxidase	3	3	3	3	3	0
Polyphenol oxidase	1	4	4	0	0	0
Pectin esterase inhibitor	1	5 (−4)	0	2	1	0
Callose synthase	0	3 (−3)	0	0	0	0

Values are the total number of DEGs from root samples at different timepoints for both cultivars. The numbers in parentheses with a “−” indicate the downregulated DEGs, if present, among all DEGs identified from a specific genotype/timepoint

### Specific transcriptional suppression in the roots of the susceptible B.9 genotype

One of the striking observations based on the analysis of this RNA-seq dataset was the overwhelmingly over-represented DEGs with downregulated expression patterns in the B.9-48 sample, which suggested a wide-spread and systematic transcriptional suppression in susceptible B.9 roots. Those downregulated DEGs encode enzymes with annotated functions at all the steps of glycolysis (Fig. 8 and Table 1). Genes encoding enzyme functioning at the downstream of glycolysis, such as pyruvate metabolism, including “phosphoenol pyruvate carboxylase”, and “pyruvate decarboxylase” were also downregulated, indicating the suppression expanded into the downstream processes of energy generation and substrate provision. Ten identified DEGs encoding “sucrose synthase” or “sucrose synthase-like” were all downregulated in the B.9-48 sample, compared with only one gene in G.935-24.

**Fig. 9:**
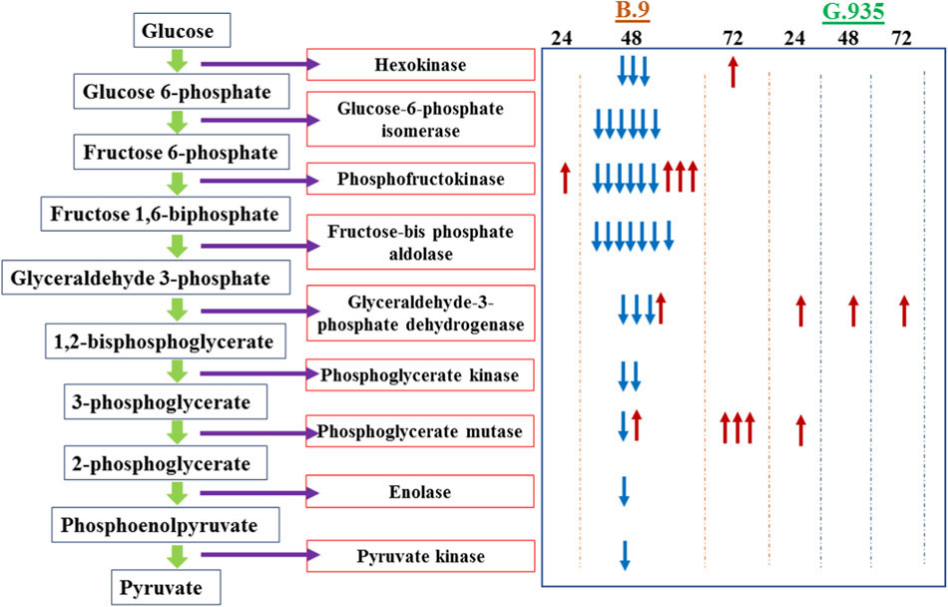
Systematic suppression of genes encoding enzymes of the glycolysis pathway in apple roots. The left panel illustrates the steps and coordinated enzymes catalyzing the reaction. The right panel indicates the identified DEGs per genotype and timepoint. Each arrow in the right panel represents an individual gene in the same gene family. Upward arrows in red color denote upregulated expression pattern; downward arrows in blue color denote downregulated expression patterns. Columns in the right panel indicate the genotypes and timepoints

DEGs encoding proteins belonging to more than a dozen functional groups were either exclusively or mostly downregulated in the B.9-48 samples. These included those (numbers of DEGs in parentheses) encoding “chaperone protein” (10), “CASP-like protein” (6), “DEAD-box ATP-dependent RNA helicase” (3), “dual specificity protein kinase” (5), G2/mitotic-specific cyclin (3), “metallothionein” (4), “glutamate decarboxylase” (6), “perakine reductase” (6), “mannitol dehydrogenase” (11), “uricase” (5), “glutamate (or aspartate, histidine, valine) − tRNA ligase” (6), and “kinesin” (4). The widespread suppression reflected the severely disturbed transcriptomes in B.9-48 due to *P. ultimum* infection. Nevertheless, no DEGs encoding these proteins were identified in G.935 (Table 1). The associations between the downregulation of these functional groups of genes with the observed susceptibility of the B.9 genotype to *P. ultimum* infection deserve future examination (Fig. 9).

### Data validation by RT-qPCR

**Fig. 10:**
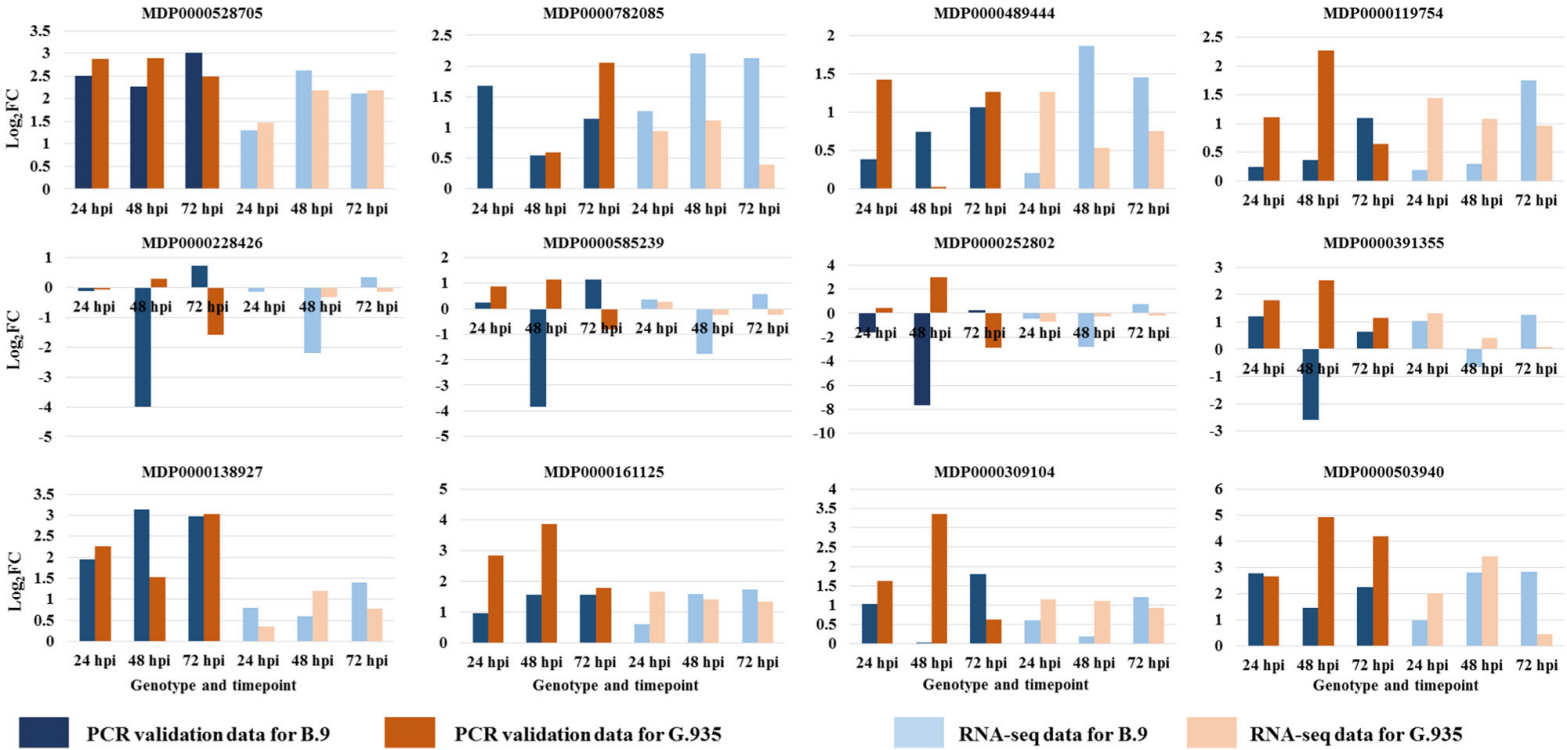
Validation of the expression patterns for genes selected from RNA-seq analysis by RT-qPCR. Gene expression values and regulation patterns were obtained by comparing between *P. ultimum* inoculated and mock-inoculation control. The values on the *Y* axis are log_2_ fold changes. The group of bars at left with darker (blue and brown) colors were values from the RT-qPCR validation; the group of bars at right with lighter blue and light brown colors represent the values from the RNA-seq dataset

The expression patterns for 12 selected genes from this dataset were validated by an independent approach of RT-qPCR (Fig. 10). Mutual agreement between RNA-seq analysis and RT-qPCR experiments was observed for a majority of these selected DEGs. Specifically, based on comparison of expression levels between control and infected tissues, about 98% of the data points (combination of genotype, target gene and timepoint) showed consistent patterns, i.e., either upregulation or downregulation, in response to *P. ultimum* infection. Gene-specific primer sets, reference numbers in the apple genome database and the value of RNA-seq data are shown in Table S1.

## Discussion

The understanding of molecular defense activation in plant roots, particularly roots of perennial tree crops, in response to infection by soilborne necrotrophic pathogens, is very limited. This study, using RNA-seq based transcriptome analysis, depicted the first panoramic picture of genotype-specific transcriptome changes in apple roots in response to *P. ultimum* infection. A recurring theme of transcriptional regulation was observed for several functional groups of genes: after a severe suppression of expression in B.9-48, the additional homologous genes from the same families were upregulated in B.9-72. Such delayed and disrupted defense activation represents a loss of opportunity for organizing an effective defense system in apple roots. One fitting example is the group of DEGs encoding “MATE efflux family protein”. As shown in Fig. 8b, four upregulated DEGs were identified in G.935-24 hpi, and none in B.9-24. Then in the B.9-48 samples, four genes were upregulated, but five others were downregulated. Until 72 hpi, four genes, which were identical to genes upregulated in G.935-24, were finally upregulated but still with two additional downregulated homologous genes. In contrast, in G.935 roots, a few genes were consistently upregulated at all three timepoints without suppression during *P. ultimum* infection. Assuming “MATE efflux family protein” plays a critical role in the timely delivery of antimicrobial metabolites to specific cellular locations for deterring pathogen progression, the delayed or interrupted induction of these genes could be one of the major limiting factors leading to the susceptibility of B.9 roots. Similarly, “sugar transporter SWEET1” and “phosphate transporter”, several gene families of JA biosynthesis enzymes, and TF-encoding genes showed similar regulation patterns of delayed induction in B.9 roots.

Early defense signaling can critically influence the outcome of plant-pathogen interactions because of the potential signal augmentation toward downstream processes. More WAK encoding genes^33,34^ were upregulated at 24 hpi in G.935 compared with B.9 roots, although a larger number of homologous genes were induced in B.9-48 and B.9-72. Most of the identified lectin RLK genes^35,36^ were upregulated during pathogenesis, but a highly-expressed MDP0000228426 was one of two downregulated lectin RLK encoding genes, which were exclusively identified in B.9-48. Among other categories of “receptor kinases”, such as “cysteine-rich receptor” and “LRR receptor-like serine/threonine protein kinases”, high ratios of downregulated members were almost always identified from the B.9-48 data subset. The MAPK signaling cascade plays a crucial role in various biotic and abiotic stress responses, through hormone-mediated induction of WRKY22 and WRKY29^37,39,45–48^. Both plant and animal pathogens are known to use their effectors to circumvent, inactivate or even ‘hijack’ MAPK-mediated defense responses^39^. A “mitogen-activated protein kinase kinase kinase 3-like” (MDP0000187103) demonstrated specific suppression at B.9-48, while in contrast, the same gene was consistently upregulated at G.935-24 and G.935-48. Disease resistance proteins function as the cytoplasmic receptors for recognizing specific pathogen effectors and activating a stronger and more specific immune response^49–51^. From the current dataset, more than three dozen of genes involved in resistance reaction were exclusively downregulated in B.9-48, indicating a stage-dependent suppression. A previous report indicated that a preformed molecular defense network may exist in pre-inoculated apple root tissues, as suggested by more abundant *R*-gene transcripts in G.935 root tissues than in B.9^26^. Such pre-existing defense systems could be critical for initiating earlier and stronger defense activation to deter pathogen attacks in G.935 roots at the early stage of infection. Both pre-existing and induced molecular defense networks likely contribute to the effective resistance responses leading to pathogen deterrence.

It has been well established that JA and ET function as the primary defense hormones in response to infection by necrotrophic pathogens^15,17,42^. In the apple root-*P. ultimum* pathosystem, the regulation of ET biosynthesis was not remarkably different between B.9 and G.935. On the other hand, a timely and effective JA biosynthesis might be one of the primary reasons leading to enhanced defense system in G.935 roots after *P. ultimum* infection. The direct connection between the robust JA biosynthesis and efficient ETI in apple roots could benefit from careful analysis in the future. The roles of TFs in the plant immune response have been well elucidated^17,19,20,22^. In the current study, DEGs encoding the members of four major TF families, WRKY, bHLH, AP2/ERF, and MYB, seemed to be targeted at transcriptional suppression in B.9 roots, particularly at 48 hpi. Similar “stage-dependent” suppression was rarely observed in G.935 roots. The delayed and interrupted defense activation was reflected by the downregulation of genes of several other functional groups, including genes for WAKs, MAPKs, and R-proteins and JA biosynthesis enzymes.

The timing and intensity of defense activation could directly impact the “chemical warfare” between plant cells and invading pathogens. The effective resistance responses require coordinated activation of a well-connected defense network without interruption. It was known that PTI-activated and ETI-activated defense responses share many features, but with different amplitude^18,51^, including callose and lignin synthesis, and the production of phytoalexins and PR proteins. Based on the observations from this dataset, the early and non-interrupted induction of genes that encode WAKs, MAPK, TFs, JA biosynthesis enzymes, and MATE efflux family proteins, may represent an effective ETI, which is operational in infected G.935 roots, while the opposite could be the case in B.9. Due to the potential functional hierarchy among these proteins, it is tempting to speculate the roles of their co-regulation patterns. For example, it was known that Arabidopsis WRKY-33 is the phosphorylation substrate for MPK3/MPK6; in turn WRKY33 directly regulates the camalexin biosynthesis^49,50^.

The effect of potential pathogenic effectors from *P. ultimum* on the widespread transcriptional suppression in B.9-48 samples are beyond the scope of the current study. Lacking efficient detoxification processes in the root cells may also account for the observed systematic suppression of multiple cellular pathways. One noticeable feature of this dataset is that a relative smaller number of DEGs was identified from G.935-72. It can be speculated that the quick and consistent defense activation at the early stage (24 and 48 hpi) may result in the sufficient deterrence of pathogen progression^28^. Therefore, the smaller portion of tissues were still experiencing active pathogenesis at 72 hpi in the resistant G.935 root.

With the longer paired-end sequence reads and three biological replicates, this comprehensive dataset offers a high-resolution global view of genotype-specific transcriptome changes associated with defense activation in apple roots during *P. ultimum* infection for the first time. The results revealed two distinct scenarios: (i) a quicker, stronger, and consistent defense response pattern in G.935 roots, and (ii) a slower, weaker, and interrupted pattern in B.9 roots. It is possible that pathogen effectors or toxins from *P. ultimum* is responsible for selective suppression of specific members in a gene family in the B.9-48 sample. Then, to compensate or recover the disrupted cellular functions, another new set of genes from the same families were activated in B.9-72. Such a delayed and/or interrupted process could be detrimental to effective defense activation, because the loss of momentum in the weakened defense system, B.9 roots were eventually overpowered by the attacking pathogen. In contrast, the earlier, stronger, and non-interrupted defense activation effectively restricts the pathogen progression in G.935 root. The ultimate defense output depends on the coordinated and synchronized activation of genes that are required to establish the successful execution of PTI and/or ETI. Results from the current study suggest that efficient and coordinated activation of several molecular components are needed for a successful resistance response, including early signal transduction (RLKS, MAPKs, and R-proteins), biosynthesis of defense hormone (JA and ET) and secondary metabolites (CHI and UGT), defense related TFs (WRKY, bHLH, ERF, and MYB), and transporters (ABC transporter and MATE efflux family protein). The results from the current study set the foundation for future hypothesis-driven studies for validating the association of these candidate genes with apple root resistance traits.

### Conclusion

This communication contributes to the unraveling of the global transcriptional networks in root cells of apple rootstock genotypes that are resistant or susceptible to *P. ultimum* infection. The comprehensive dataset from this comparative transcriptome analysis revealed contrasting scenarios between these two genotypes. The more dramatically disturbed transcriptomes in the roots of the susceptible genotype B.9 reflected an overwhelmingly larger number of downregulated DEGs at 48 hpi, a critical stage for this pathosystem. In contrast, a majority of the DEGs were upregulated in the less agitated G.935 root transcriptome. Several groups of genes encoding proteins functioning at key steps of defense activation were often readily upregulated at 24 dpi in roots of G.935, but the upregulation of their homologues was often delayed until 72 hpi in B.9 roots, likely due to the severe suppression at 48 hpi. Such genes included those encoding kinase receptors, MAPK, JA biosynthesis enzymes, TFs, UGTs, secondary metabolite biosynthesis and transporters. These observations indicated that a quicker, stronger, and more consistent defense activation created an effective ETI and/or efficient detoxification capacity in the roots of G.935, inhibiting the progression of necrosis caused by *P. ultimum*. In contrast, lack of an effective ETI and inability to detoxify the pathogen’s phytotoxins due to delayed and interrupted defense responses, could have resulted in the observed vulnerability of susceptible B.9 roots. The systematic suppression of the primary metabolism in susceptible B.9 roots at the key stage of 48 hpi, including glycolysis and many other cellular processes, disrupted the supply of cellular energy and metabolic precursors. Detailed understanding of the mechanisms behind such wide-spread suppression is still largely elusive, but a possible explanation may be the existence of a susceptibility gene in B.9, but not G.935. The identified DEGs from this study are valuable source of candidate genes for investigating their association with resistance traits among a wider selection of apple rootstock germplasm.

## Materials and methods

### Preparation of apple plants by tissue culture procedure

Tissue culture based micro-propagation procedures were used to obtain individual apple plants for both B.9 and G.935 apple rootstock genotypes as described previously^28^, as it is impossible to produce genetically uniform apple plants by seed germination. A synchronized micro-propagation process was carried out to generate plants with non-contaminated root tissues at equivalent developmental stages for both genotypes.

After root elongation providing a sufficiently large root system, plants were transferred into pots containing autoclaved Sunshine^TM^ potting mix (SUN GRO Horticulture Ltd, Bellevue, WA) for in-soil acclimation of the roots for one week before *P. ultimum* inoculation^28^. To minimize transplanting effects from tissue culture medium to soil conditions, a transparent 7” Vented Humidity Dome (Greenhouse Megastore, Danville, IL) was placed on top of a 10 × 20-inch flat tray holding the pots for retaining humidity. An identical watering schedule of every other day was applied to all plants.

### Inoculum preparation, infection process, and tissue collection

The *P. ultimum* isolate used in this study originated from the roots of ‘Gala’/M26 apple grown in Moxee, WA, USA^4^. The procedures of inoculum preparation, quantification, and root-dip inoculation were as described previously^28^. After one week in-soil acclimation of the root system, inoculation was carried out by dipping the roots in an inoculum solution for 5 s, and inoculated plants were immediately transplanted into soil-containing pots in the format of three plants per 4” diameter pot. Root tissues from mock-inoculated and *P. ultimum* inoculated plants for both B.9 and G.935 genotypes were collected as pooled root tissues from three individual plants at designated timepoints according to the experimental design. A total of 36 samples consisted of those for two genotypes with two treatments (mock-inoculated and *P. ultimum* inoculated) and three biological replicates at three timepoints (24, 48, 72 dpi). The experimental design of focusing on 24, 48, and 72 hpi was based on an earlier transcriptome survey on apple root-Pythium interaction^27^. At designated timepoints, roots from three plants in a pot were carefully excavated from the soil, rinsed under running tap water, separated from aboveground tissues and flash frozen using liquid nitrogen and stored in −80 °C freezer until RNA isolation.

### Total RNA isolation and high-throughput mRNA sequencing

Total RNA isolation was followed the method previously described by Zhu et al.^28^. Root tissues of both resistant G.935 and susceptible B.9 were represented by three biological replicates, and each replicate included the pooled root tissues from three plants. The frozen root tissue samples were ground to a fine powder in liquid nitrogen, and RNA quantity was determined using a Nanodrop spectrophotometer (ND-1000, Thermo Fisher Scientific). The RNA integrity number (RIN) was evaluated using an Agilent 2100 Bioanalyzer. Only RNA with a RIN value of *x* ≥ 8 was used for RNA-seq. The library preparation and RNA-sequencing with 150 bp paired-end (PE) were completed at the Center for Genome Research and Biocomputing in Oregon State University using an Illumina HiSeq^TM^ 3000 (Illumina Inc., San Diego, CA, USA).

### Mapping of sequence reads and DEG analysis

Reads from B.9 (susceptible) and G.935 (resistant) libraries were mapped to the nucleotide sequences of predicted coding genes of the *Malus* x *domestica* Whole Genome v3.0.a1 (https://www.rosaceae.org/ analysis/162) using the ultrafast, memory-efficient short read aligner Bowtie2-2.2.5, which utilizes a Burrows–Wheeler index^52^. Count data were obtained for each coding sequence. Each library had three biological replicates. The DESeq2 program in R (http://www.r-project.org/) performed normalization using geometric mean and the median to normalize the data. For each comparison, the geometric mean was calculated for each gene across all samples. The counts for a gene in each sample was then divided by the geometric mean. The median of these ratios in a sample was the calculated size factor for that sample. This size factor was used to correct for library size or sampling depth and composition bias of the RNA sample. After size factors were calculated to normalize the data, the estimate of dispersion is determined. DESeq2 then used a negative binomial GLM fitting and Wald statistic in the determination of DEGs, where the *p*-value from the Wald Test indicates the probability that the observed difference between treatment and control is observed. The adjusted value (*p*-adj) was calculated using the Benjamin–Hochberg correction. The *p*-adjusted value was similar to the false discovery rate. The *p*-adjusted value < 0.05 is the preferred value used in determining statistically significant deferentially expressed genes^53^.

DEGs were identified by comparing transcript abundance between mock-inoculated control root tissues and those from *P. ultimum* infected root tissues with the cut-off values of Log_2_Fold Change ≥ 1 and the *p*-adj (adjusted *p*) values ≤ 0.05. The annotation of these genes was carried out by BLASTP^54^ against NR (non-redundant protein sequences) database, and a BLAST database containing genomic sequences for Arabidopsis (*Arabidopsis thaliana*), corn (*Zea mays*), *Medicago truncatula*, rice (*Oryza sativa*), and tomato (*Solanum lycopersicum*). For concise description, a subset of DEGs from a specific sub-dataset (genotype/timepoint), terms such as B.9-48 or G.935-24 were used to represent the specific combination of genotype and timepoint. BioVenn^55^ was used to determine the variation of the DEG inventories between two timepoints.

### Validation of the expression pattern for selected DEGs by qRT-PCR

The same total RNAs that were used for RNA-seq experiments were also used for RNA-seq data validation by qRT-PCR. The total RNA was treated with DNase I (Qiagen, Valencia, CA) and then purified with RNeasy cleanup columns (Qiagen, Valencia, CA). Two microgram of DNase-treated RNA was used to synthesize first-strand cDNA using SuperScript^TM^ II reverse transcriptase (Invitrogen, Grand Island, NY) and poly dT (Operon, Huntsville, AL) as the primer. The RT-qPCR procedure was performed as previously reported^26^. The target gene expression was normalized to that of a previously validated internal reference gene (MDP0000095375) specific for gene expression analysis in apple roots^56^ using the 2^−∆∆C^^T^ method (the comparative Ct method)^57^.

## Electronic supplementary material

Supplementary materials
