# Supplementary material for: Genotype-specific suppression of multiple defense pathways in apple root during infection by Pythium ultimum
Source: Hortic Res. 2019 Jan 1;6:10. doi: 10.1038/s41438-018-0087-1 (PMC6312547; doi:10.1038/s41438-018-0087-1)
Supplement: Supplementary file 1 — Supplementary materials [file 41438_2018_87_MOESM1_ESM.docx]

**Additional files**


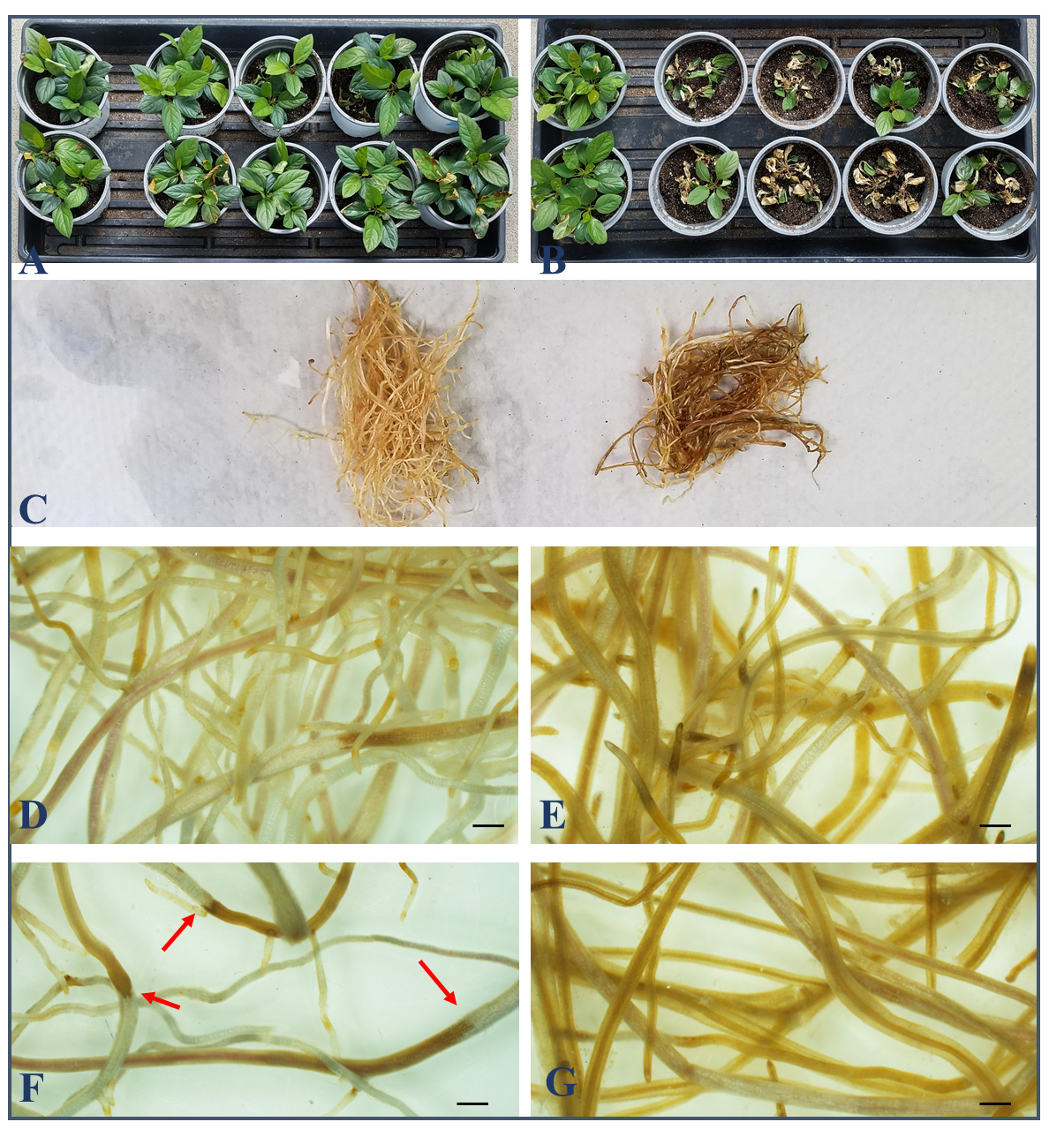


**Figure S1**. Phenotypes for B.9 and G.935 in response to infection by P. ultimum. **A.** Survival rate of G.935 plants at 14 dpi (days post inoculation), six plants at left side of the tray are mock inoculated plants as control. **B.** Survival rate of B.9 plants at 14 dpi, six plants at left side of the tray are mock inoculated plants as control. **C.** Roots from infected plant at 14 dpi, left: roots infected G.935 plants; right: roots infected B.9 plants. D and F. images of infected roots from G.935 under microscope. E and G. images of infected roots from G.935 under microscope. The brown or yellow color represent the necrotic roots from *P. ultimum* infection; roots in white color. Clear line between healthy and necrotic tissue were frequently observed in the infected roots of resistant G.935 genotype. Bar represent 200 µ.


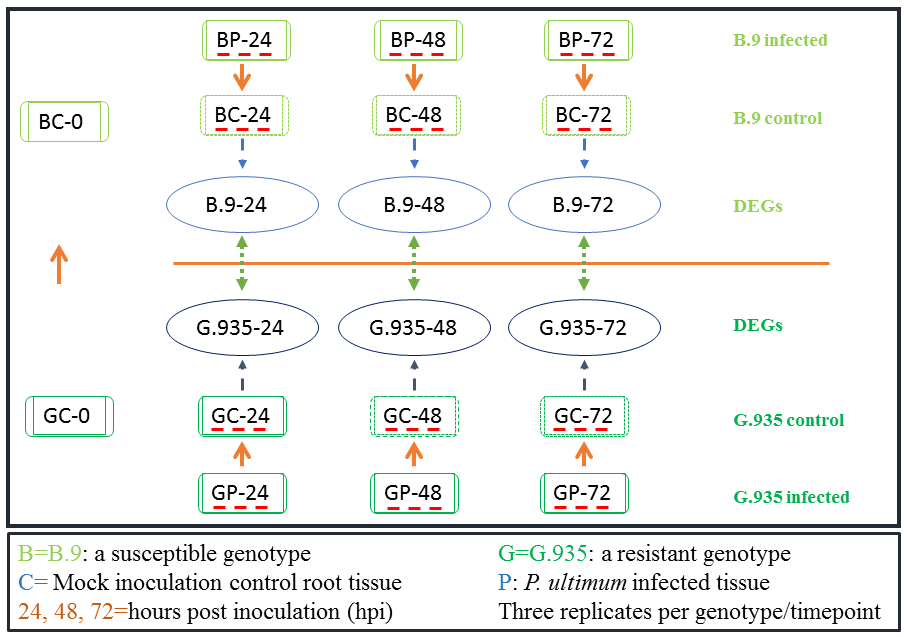


**Figure S2**. Experimental design for comparing transcriptome changes.

Plant from two genotypes were prepared by the synchronized tissue culture based micropropagation procedure, so plants were with equivalent age. The root-dip inoculation procedure was carried out simultaneously for both genotypes.

Supplemental Table S1. DEGs with annotated function in biosynthesis and signaling of other plant hormones

| Function annotation/  Gene model | Log_2_FC per genotype and timepoint | | | | | |
| --- | --- | --- | --- | --- | --- | --- |
|  | B.9-24 | B.9-48 | B.9-72 | G.935-24 | G.935-48 | G.935-72 |
| **Indole-3-acetic acid-amido synthetase GH3.1** | | | | | |  |
| MDP0000121609 |  | 2.15 |  |  |  |  |
| MDP0000209432 |  | 1.42 |  |  | 1.09 |  |
| MDP0000612660 | 1.02 | 2.36 |  |  | 1.45 |  |
| MDP0000568498 |  |  | -1.17 |  |  |  |
| **IAA-amino acid hydrolase ILR1-like** | | | | | | |
| MDP0000319659 |  | 1.23 | 1.36 |  |  |  |
| MDP0000491020 |  | 1.80 | 1.00 |  |  |  |
| MDP0000454027 |  | 1.59 | 1.12 |  |  |  |
| MDP0000249564 |  | 2.02 | 1.12 |  | 1.22 |  |
| **Indole-3-acetic acid-induced protein ARG2** | | | | | | |
| MDP0000564193 |  | -1.93 |  |  |  |  |
| MDP0000269907 |  | -2.13 |  |  |  |  |
| MDP0000385497 |  | -2.38 |  |  |  |  |
| **Auxin-responsive protein IAA1** | | | | | | |
| MDP0000123816 |  | -2.93 |  |  |  |  |
| MDP0000253285 |  | -3.11 |  |  |  |  |
| MDP0000663301 |  | -1.96 |  |  |  |  |
| MDP0000232116 |  | -1.72 |  |  |  |  |
| **Auxin-binding protein ABP19a** | | | | | | |
| MDP0000314286 | 1.4 | 2.5 | 1.9 | 1.1 | 1.2 |  |
| **Cytokinin hydroxylase** | | | | | | |
| MDP0000290662 | 2.37 | 3.17 | 3.68 | 1.34 | 1.75 |  |
| MDP0000206482 |  | 4.85 | 1.66 |  | 1.19 |  |
| MDP0000305091 | 3.89 | 4.39 | 4.40 | 1.15 | 2.06 |  |
| **Cytokinin dehydrogenase** | | | | | | |
| MDP0000271354 |  | 2.43 | 1.64 | 1.1 |  |  |
| **Strigolactone esterase DAD2** | | | | | | |
| MDP0000888050 |  | 1.34 |  |  |  |  |
| MDP0000898597 |  | 1.29 |  |  |  |  |
| MDP0000172849 |  | 1.30 |  |  |  |  |
| MDP0000529739 |  | 1.63 |  |  |  |  |
| **Carotenoid cleavage dioxygenase** | | | | | | |
| MDP0000555220 | 1.03 |  |  |  |  |  |
| MDP0000227870 |  |  |  | -1.49 |  |  |
| **Gibberellin 20 oxidase** | | | | | | |
| MDP0000245720 | 2.44 | 3.10 | 2.44 | 3.01 | 3.49 |  |
| **Abscisic acid 8'-hydroxylase** | | | | | | |
| MDP0000166337 |  |  |  | -1.47 | -1.22 |  |
| MDP0000037814 |  | 2.87 |  |  |  |  |

Numbers represent the Log_2_FC Values of gene expression for individual gene models in infected tissues after normalized by the values from corresponding mock inoculated control tissues. + stands for upregulated

Supplementary Table S2. Primer sequences used for data validation by qRT-PCR

| Gene Number | Forward primer (5’-3’) | Reverse primer (5’-3’) |
| --- | --- | --- |
| MDP0000528705 | CGGATTATGTACCAGAGACCTTAC | CACTGCCCTTGACTCTGTT |
| MDP0000782085 | CAGCAAGCTCTGTGTTGTTTG | GCGCCATTCGAGGGATTTA |
| MDP0000489444 | ATGACAAGGAAGAAGGGAACAA | AGTGGTTTCAAGGAACGGATAA |
| MDP0000119754 | GCAGAGTTGTCTTAGCACCAT | AGTGTCTGGGTACCTTCCTT |
| MDP0000228426 | GAATGCCAGCTTCAGAGTTAGA | TCCGATCCCTTTCTCTTGTTTC |
| MDP0000585239 | CTTCCGCTGCTTACGGTATTAG | GGCAACTCAACAACAAGAGAATG |
| MDP0000252802 | GGTGGCCAGGTTGTCTATATC | TGAATGCTCACACCCGTAAA |
| MDP0000391355 | CGTCCAAGTTGTTCAGGTCTTA | CAAGCACCAAGAGTGTCATTTC |
| MDP0000138927 | GAACGAAGGGCACTCAAAGA | GCCACATAGAAACAAGACCAAAG |
| MDP0000161125 | CAGTACAACATGCTCGGGATAC | AGCACAGGGATTACCCATAGA |
| MDP0000309104 | GTTCTTCAGGGACATCGTTACA | CTATATCGGCAACTGAGGAAGG |
| MDP0000503940 | GACTGTGAAGCCTGTTCCTAAT | CAT CAA ATC ACTTTTTGGGTTG |

This file contains the sequences 12 primer pairs that were used to validate RNA-seq data by RT-qPCR methods.
